# The impact of CCR8+ regulatory T cells on cytotoxic T cell function in human lung cancer

**DOI:** 10.1038/s41598-022-09458-5

**Published:** 2022-03-30

**Authors:** Miya Haruna, Azumi Ueyama, Yoko Yamamoto, Michinari Hirata, Kumiko Goto, Hiroshi Yoshida, Naoko Higuchi, Tetsuya Yoshida, Yujiro Kidani, Yamami Nakamura, Morio Nagira, Atsunari Kawashima, Kota Iwahori, Yasushi Shintani, Naganari Ohkura, Hisashi Wada

**Affiliations:** 1 Department of Clinical Research in Tumor Immunology, Graduate School of Medicine, Osaka University, Osaka, 565-0871 Japan; 2 Pharmaceutical Research Division, Shionogi & Co., Ltd., Osaka, 561-0825 Japan; 3 Department of Basic Research in Tumor Immunology, Graduate School of Medicine, Osaka University, Osaka, 565-0871 Japan; 4 Department of Experimental Immunology, Immunology Frontier Research Center, Osaka University, Osaka, 565-0871 Japan; 5 Department of Urology, Graduate School of Medicine, Osaka University, Osaka, 565-0871 Japan; 6 Department of General Thoracic Surgery, Graduate School of Medicine, Osaka University, Osaka, 565-0871 Japan

**Keywords:** Immunology, Immunotherapy, Lung cancer, Tumour immunology

## Abstract

Regulatory T cells (Tregs) suppress the host immune response and maintain immune homeostasis. Tregs also promote cancer progression and are involved in resistance to immune checkpoint inhibitor treatments. Recent studies identified selective CCR8 expression on tumor-infiltrating Tregs; CCR8+ Tregs have been indicated as a possible new target of cancer immunotherapy. Here, we investigated the features of CCR8+ Tregs in lung cancer patients. CCR8+ Tregs were highly activated and infiltration of CCR8+ Tregs in tumors was associated with poor prognosis in lung cancer patients. We also investigated their immune suppressive function, especially the influence on cytotoxic T lymphocyte cell function. The Cancer Genome Atlas analysis revealed that CD8 T cell activities were suppressed in high CCR8-expressing tumors. Additionally, depletion of CCR8+ cells enhanced CD8 T cell function in an ex vivo culture of lung tumor-infiltrating cells. Moreover, CCR8+ Tregs, but not CCR8− Tregs, induced from human PBMCs markedly suppressed CD8 T cell cytotoxicity. Finally, we demonstrated the therapeutic effect of targeting CCR8 in a murine model of lung cancer. These findings reveal the significance of CCR8+ Tregs for immunosuppression in lung cancer, especially via cytotoxic T lymphocyte cell suppression, and suggest the potential value of CCR8-targeted therapy for cancer treatment.

## Introduction

Lung cancer is the most common malignancy and the major cause of cancer death worldwide^1^. About 85% of lung cancers are non-small-cell lung cancer (NSCLC), which includes adenocarcinoma, squamous-cell carcinoma, and large-cell carcinoma^2^. Immune checkpoint therapy, such as anti-PD-1 or PD-L1 antibody therapy, has significantly improved survival in lung cancer patients^3–5^. These treatments restore the anti-tumor activity of T cells and relieve tumor-mediated immunosuppression^6^. However, some patients do not respond to immune checkpoint inhibitors, and the complete response rate is not sufficient. One possible cause of the non-response to immune checkpoint inhibitor therapy is the infiltration of immunosuppressive cells in tumors, such as the regulatory T cells (Tregs)^7,8^.

Tregs are a subgroup of the CD4 T cell population that is defined by sustained FOXP3 expression. Tregs suppress the activation of immune cells and maintain immune homeostasis to protect the host against the development of allergic and autoimmune diseases. Tregs also enhance cancer progression by suppressing the anti-tumor immune responses of effector immune cells, mainly cytotoxic T lymphocytes (CTLs)^9,10^. CTLs exhibit anti-cancer activity by playing important roles in granule exocytosis and Fas-mediated cytotoxicity. CTLs also release several cytokines such as interferon-γ (IFNγ) to induce cancer growth arrest^11^. Tregs provide an immunologic barrier against these CTL-mediated anti-tumor immune responses. The ratio of Tregs to CD8 T cells in the tumor microenvironment is associated with a poor prognosis, which suggests that Tregs suppress the tumor antigen-specific T cell activities^12^. Tregs have been identified in multiple types of malignant lesions including head and neck, breast, lung, pancreas and skin cancers, leukemia, and malignant lymphoma^13^. Therefore, the depletion of Tregs or the control of Treg function may represent promising immunotherapeutic strategies for cancer treatment.

Multiple research groups have been exploring the development of Treg-targeted therapies. Mouse antibodies against CTLA-4, CD25, OX-40, and glucocorticoid-induced TNF receptor (GITR) effectively abrogated tumor growth by antibody-dependent cellular cytotoxicity–mediated Treg depletion in a murine model of cancer^8^. A monoclonal antibody against C–C chemokine receptor 4 (CCR4) is one of the Treg-targeted treatments in clinical trials for human cancer and was shown to efficiently deplete Tregs in peripheral blood^14^. However, some issues still need to be addressed before clinical application of these treatments. One issue is how to circumvent the deleterious autoimmunity that may accompany the depletion of Tregs. In addition, these Treg-targeted therapies using antibodies targeting the above molecules can result in the depletion of tumor-reactive effector T cells, leading to the reduction of anti-tumor immunity^15^. Therefore, it is critically important to develop a strategy that only removes tumor-infiltrating Tregs.

C–C chemokine receptor 8 (CCR8) is a chemokine receptor, whose ligands are CCL1, CCL8, and CCL18^16^. Recent studies reported selective CCR8 expression on tumor-infiltrating Tregs^17–23^. In some murine models, CCR8+ Tregs highly express Treg-related molecules that are associated with suppressive functions, and anti-CCR8 antibody treatment reduces tumor volume and enhances anti-tumor immunity^17,18^. Our previous study using murine tumor models of colorectal cancer (CT26) and breast cancer (EMT6) was in line with these findings^19^. In humans, increased CCR8 expression in tumors was related to poor patient prognosis in several types of cancer^21–23^ and the correlation of CCR8+ Treg infiltration and low function of T cells in tumors was also reported in bladder cancer^23^. These findings indicate that anti-CCR8 antibody might represent a potential cancer treatment. However, to the best of our knowledge, the impact of CCR8+ Tregs on CTL function in human cancer is not fully understood yet.

In this study, we examined the involvement of CCR8+ Tregs in human lung cancer and their functional profiles. We demonstrated that CCR8+ Tregs were highly suppressive against CTL functions, but CCR8− Tregs were not. The data suggest the potential impact of CCR8+ Tregs on cancer immunity and the potential of CCR8-targeted therapy for lung cancer immunotherapy.

## Results

### CCR8 is selectively expressed on tumor-infiltrating Tregs in human lung cancer

**Figure 1:**
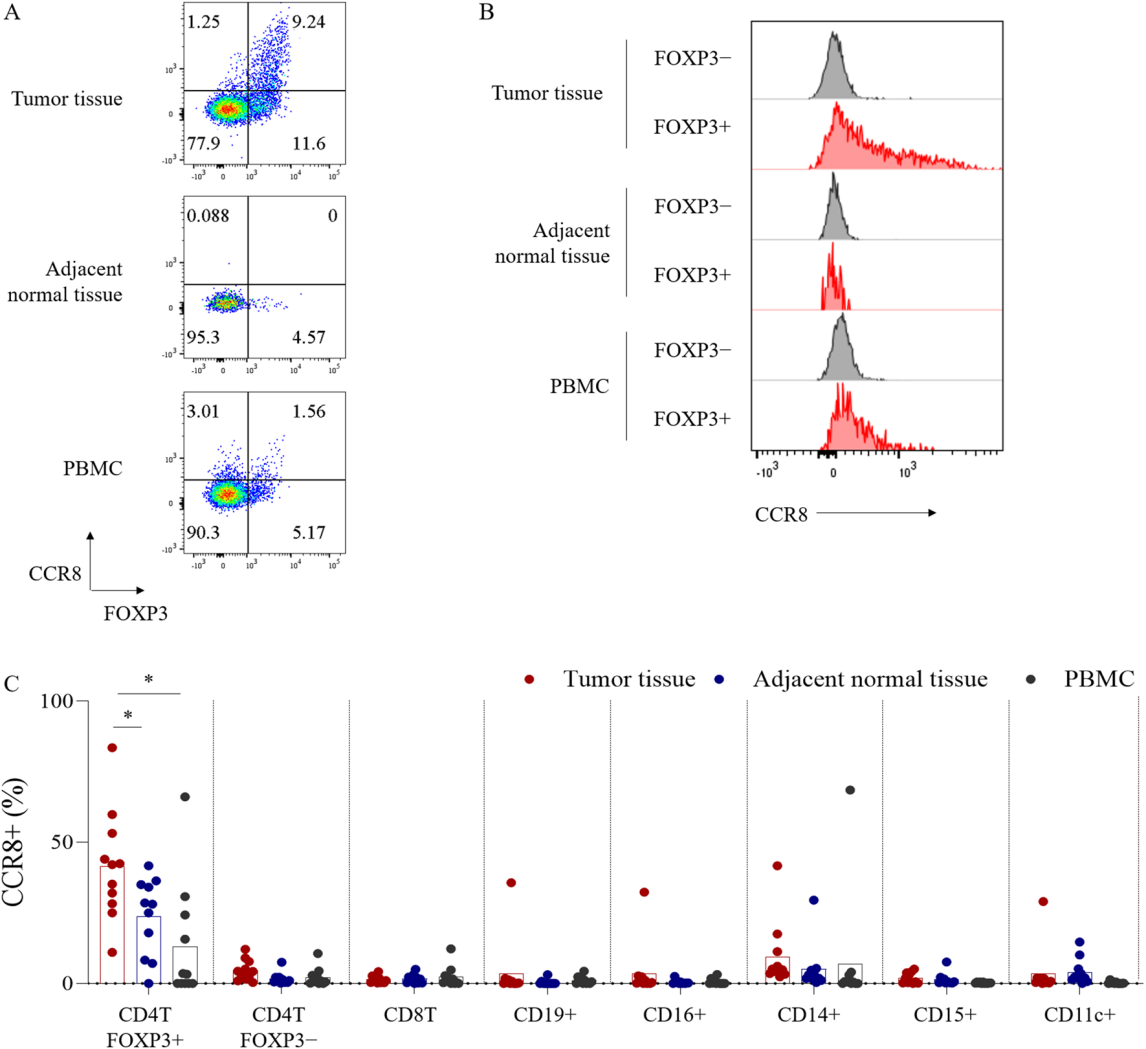
CCR8 expression on immune cells of lung cancer patients. Single cell suspensions isolated from lung tumor tissues, tumor-adjacent normal tissues and PBMCs were analyzed by flow cytometry. (**A**) FOXP3 and CCR8 expressions on CD4 T cells (gated on live CD45+ CD3+ CD4+ cells). (**B**) CCR8 expression on FOXP3+ or FOXP3− CD4 T cells. Representative flow cytometric data are shown. (**C**) Percentages of CCR8+ cells in FOXP3+ CD4 T, FOXP3− CD4 T, CD8 T, CD19+, CD16+, CD14+, CD15+, and CD11c+ cell subsets in lung tumors, tumor-adjacent normal tissues and PBMCs (n = 11). Data are shown as mean values ± SD. Statistical significance was determined by paired t-test (*p ≤ 0.05; **p < 0.01) compared with tumor infiltrating FOXP3+ CD4 T cell group.

We first analyzed CCR8 expression on immune cells obtained from 11 lung cancer patients by flow cytometry. We detected more FOXP3+ Tregs in tumor tissues than in adjacent normal tissues, Peripheral blood mononuclear cells (PBMCs) and lymph nodes (Fig. 1A, Supplementary Fig. S1). CCR8 expression level in Tregs was highest in tumor tissues and higher than in FOXP3− conventional CD4 T cells (conv CD4 T cells) (Fig. 1A, B). The frequency of CCR8+ cells in other immune-related cell populations was low (Fig. 1C).

### CCR8+ Treg infiltration is associated with poor prognosis in lung cancer

**Figure 2:**
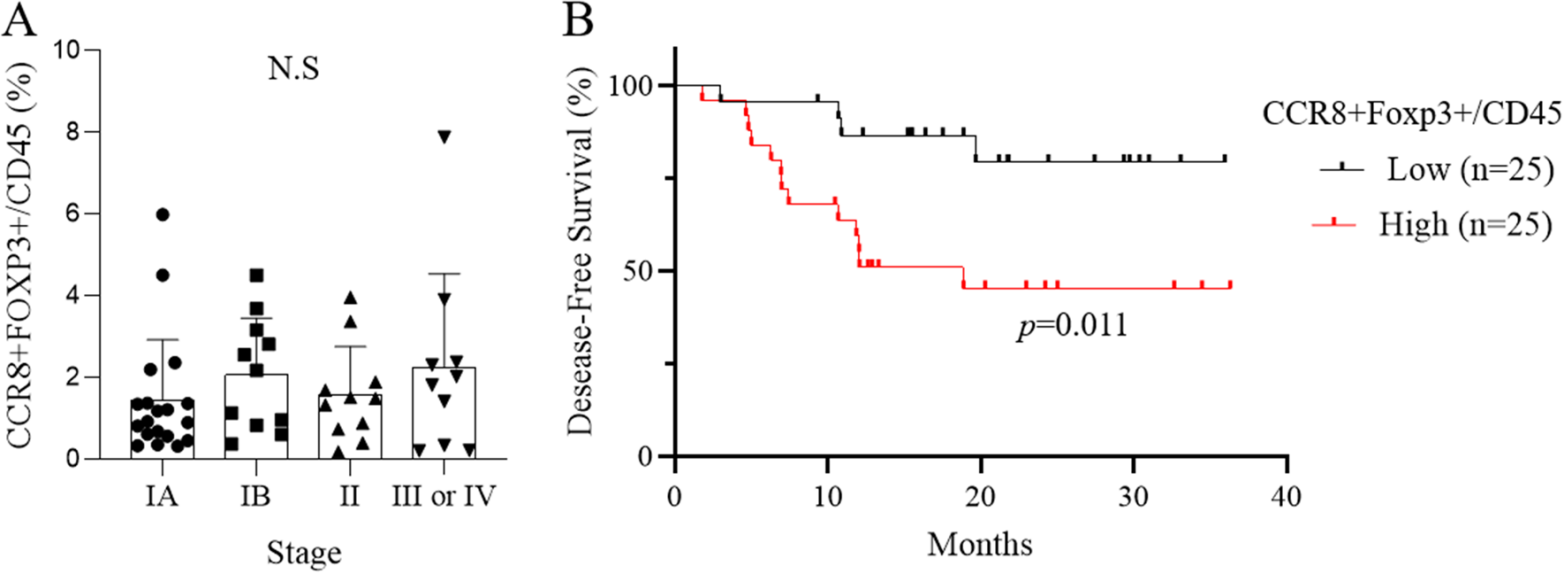
Frequency of CCR8+ Tregs is associated with patient prognosis but not tumor stage. (**A**) Percentages of CCR8+ FOXP3+ cells in CD45+ cells in lung tumors based on clinical stage (n = 50). Statistical significance was determined by one-way ANOVA Kruskal–Wallis test followed by Dunn. (**B**) Kaplan–Meier analysis of disease-free survival based on the level of CCR8+ Tregs. Lung cancer patients (n = 50) were divided into high and low CCR8+ Treg groups based on the median percentage of CCR8+ FOXP3+/CD45+ cells in TICs. Statistical significance was determined by the log-rank (Mantel–Cox) test.

To determine the clinical significance of CCR8+ Tregs in lung cancer, we evaluated the frequency of CCR8+ Tregs in tumor-infiltrating CD45+ cells in 50 lung cancer patients by flow cytometry and analyzed the correlation with clinical stage and outcome. There was no significant relationship between CCR8+ Treg infiltration and clinical cancer stage (Fig. 2A). We next divided 50 lung cancer patients into the CCR8-high and low groups based on the median value of the percentage of CCR8+ Tregs in CD45+ cells and found that the disease-free survival (DFS) of the CCR8-high group was significantly shorter than that of the CCR8-low group (*p* = 0.011) (Fig. 2B). The clinical data of both groups are shown in Supplementary Table 1. There was no significant difference in characteristics between the CCR8-high group and CCR8-low group except for age.

### Expression profiles of CCR8+ Tregs in lung cancer

**Figure 3:**
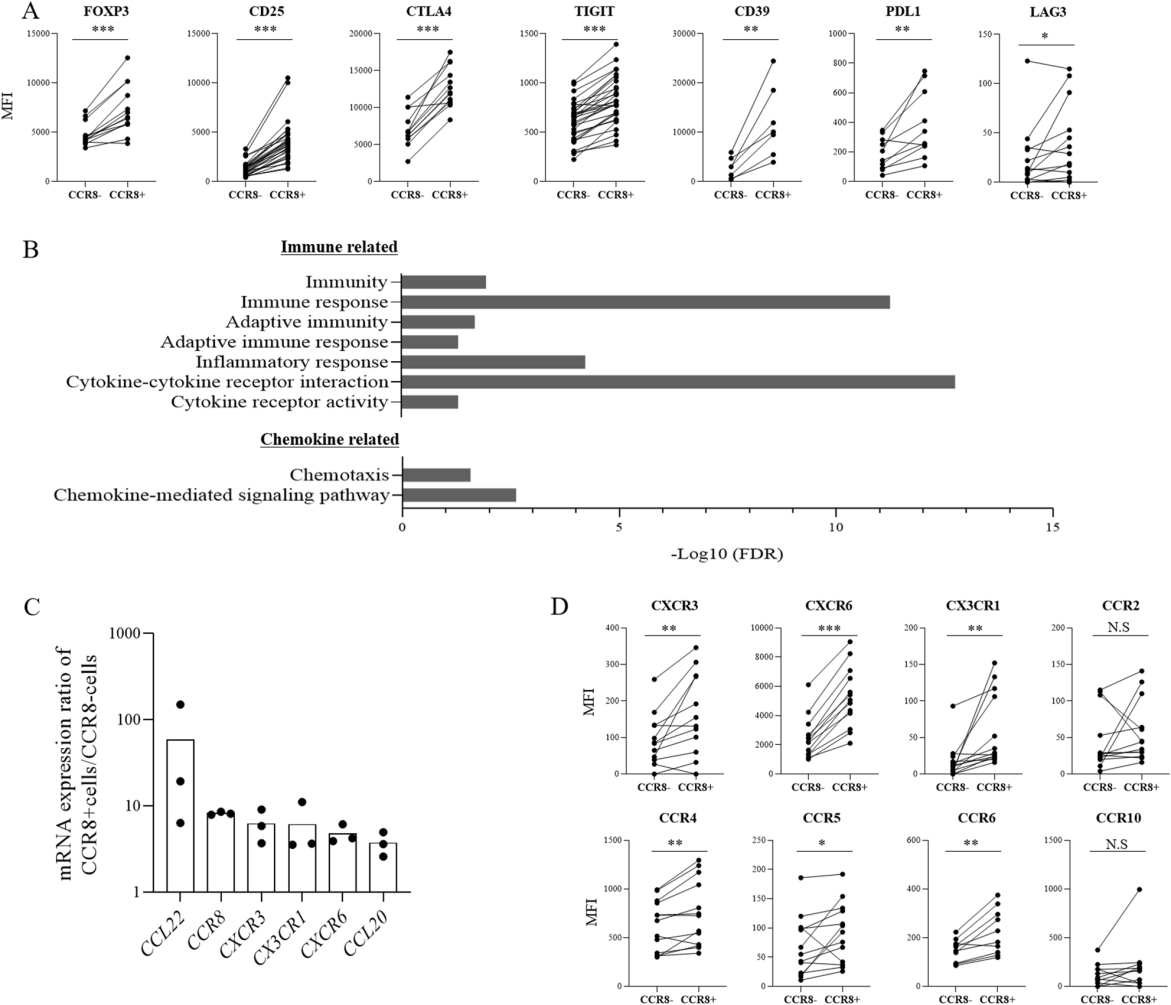
Characteristics of lung tumor–infiltrating CCR8+ and CCR8− Tregs. (**A**) Protein expression levels of Treg-related molecules were analyzed by flow cytometry (n = 7–22). The mean fluorescence intensity (MFI) of each stained molecule on CCR8− Tregs and CCR8+ Tregs (gated on live CD45+ CD3+ CD4+ Foxp3+ cells) is shown. Lines connect data from the same donor samples. (**B**) CCR8+ Tregs and CCR8− Tregs (gated on live CD45+ CD3+ CD4+ CD25^high^ cells) were sorted from lung TICs obtained from 3 patients. RNA-sequencing was performed and differentially expressed genes (DEGs) between CCR8+ Tregs and CCR8− Tregs were identified. Gene Ontology (GO) enrichment analysis of the top 100 upregulated DEGs in CCR8+ Tregs sorted by p-value was performed using the DAVID bioinformatics tool. Enriched immune-related terms categorized in biological process were extracted. The minus logarithm of False Discovery Rate (FDR) is shown. (**C**) mRNA expressions of chemokine-related genes were analyzed and the ratios of mRNA expression in CCR8+ Tregs to that in CCR8− Tregs are shown (n = 3). (**D**) Protein expression levels of chemokine receptors were detected by flow cytometry. The MFI of each stained molecule on CCR8− and CCR8+ Tregs (gated on live CD45+ CD3+ CD4+ Foxp3+ cells) is shown (n = 10–13). Lines connect data from the same donor samples. Statistical significance was determined by paired t-test (*p ≤ 0.05; **p < 0.01; ***p < 0.001).

We next analyzed the profile of CCR8+ Tregs in lung tumors. Treg-related molecules that are associated with suppressive functions, such as FOXP3, CD25, CTLA4 and CD39, were highly expressed in CCR8+ Tregs compared with CCR8− Tregs as confirmed by flow cytometry (n = 7–22) (Fig. 3A, Supplementary Fig. 2). We performed RNA-sequencing of CCR8+ Tregs and CCR8− Tregs sorted from tumor-infiltrating cells (TICs) obtained from 3 patients and identified differentially expressed genes (DEGs) between CCR8+ Tregs and CCR8− Tregs. Gene Ontology (GO) enrichment analysis of the top 100 DEGs with the lowest p-value among the upregulated genes in CCR8+ Tregs was performed using DAVID. The enriched immune-related terms categorized in biological process are shown in Fig. 3B. The upregulated genes in CCR8+ Tregs were mainly associated with adapted immunity and inflammatory signaling. Furthermore, the chemokine-related pathway was also enriched. Significantly upregulated chemokine genes (p < 0.05) in CCR8+ Tregs are shown in Fig. 3C (n = 3). We then analyzed the expression of chemokine receptors by flow cytometry (n = 10–13) and found that CCR8+ Tregs expressed higher levels of various chemokine receptors compared with levels in CCR8− Tregs (Fig. 3D).

### CD8 T cell activities are suppressed in high CCR8-expressing tumors

**Figure 4:**
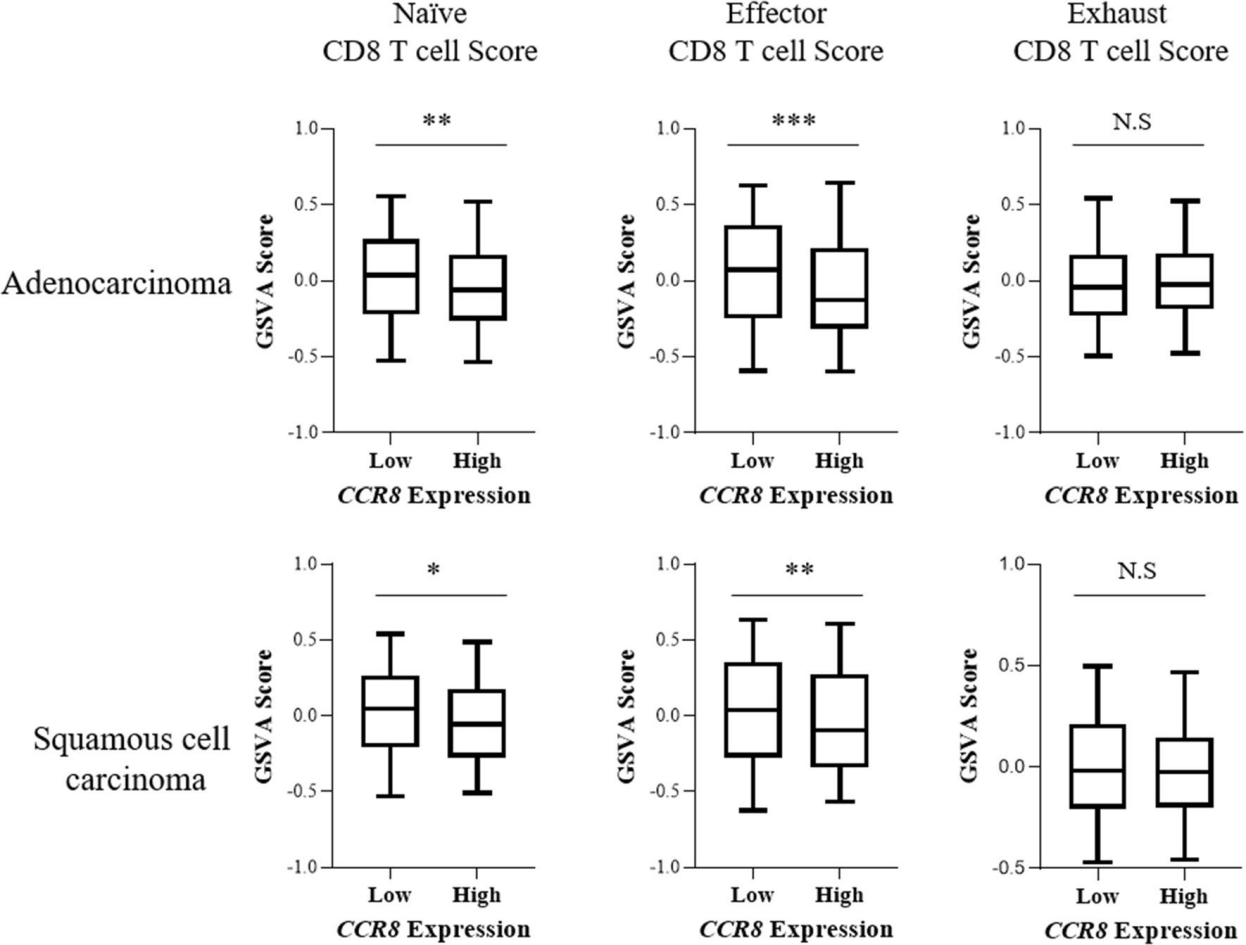
Naïve, effector and exhausted CD8 T cell enrichment scores in CCR8-high and CCR8-low lung tumors. Whole tumor mRNA expression data of patients with adenocarcinoma (n = 510) and squamous cell carcinoma (n = 484) were obtained from TCGA cBioPortal database. Patients were divided into high and low CCR8 groups based on the median *CCR8*/*PTPRC* ratio. Gene Set Variation Analysis (GSVA) was performed with naïve, effector and exhausted CD8 T cell signature genes, as described by Guo et al*.*, and the calculated GSVA enrichment scores in CCR8-high and low group are shown. Statistical analysis significance was determined by unpaired t-test with Welch’s correction (*p ≤ 0.05; **p < 0.01; ***p < 0.001).

Because CCR8+ Tregs highly express Treg-related molecules that are associated with suppressive functions, we hypothesized that tumors with high CCR8 expression might exhibit a suppressed immune profile. To investigate this hypothesis, we examined mRNA expression data of lung adenocarcinoma (n = 510) and lung squamous cell carcinoma (n = 484) obtained from the cBioPortal The Cancer Genome Atlas (TCGA) database. We found a correlation between the gene expression of *CCR8* and *receptor-type tyrosine-protein phosphatase C* (*PTPRC*), which encodes the lymphocyte marker CD45 (Supplementary Fig. S3). To estimate the contribution of CCR8-expressing cells to other immune cells, we calculated the *CCR8*/*PTPRC* expression ratio and divided patients into the CCR8-high and low groups based on the median value of the *CCR8*/*PTPRC* ratio. We then examined the differences of immune-related gene expression between the two groups. We found that gene related T cell and antigen-presenting cell (APC) were significantly reduced in CCR8-high tumors compared with levels in CCR8-low tumors in both lung adenocarcinoma and lung squamous cell carcinoma (Supplementary Table S2). Because CTLs are main players in anti-cancer immunity, we focused on CD8 T cells. To characterize gene expression signatures and pathway activation associated with CD8 T cell function, we performed Gene Set Variation Analysis (GSVA) with selected gene sets from the literature^24^. According to Guo et al.^24^, the CD8-C1-LEF1 fraction, CD8-C3-CXCR1 fraction and CD8-C6-LAYN fraction were used as naïve, effector and exhausted CD8 T cell gene sets, respectively. Naïve, effector, and exhausted CD8 T cell scores were calculated by GSVA, and we compared the scores between CCR8-high and -low tumors (Fig. 4). The CCR8-high group showed significantly lower naïve and effector CD8 T cell scores compared with the CCR8-low group. In particular, the difference in the effector CD8 T cell score between the two groups was remarkable in both lung squamous cell carcinoma and lung adenocarcinoma.

### Depletion of CCR8+ cells enhances CD8 T cell function in lung tumors

**Figure 5:**
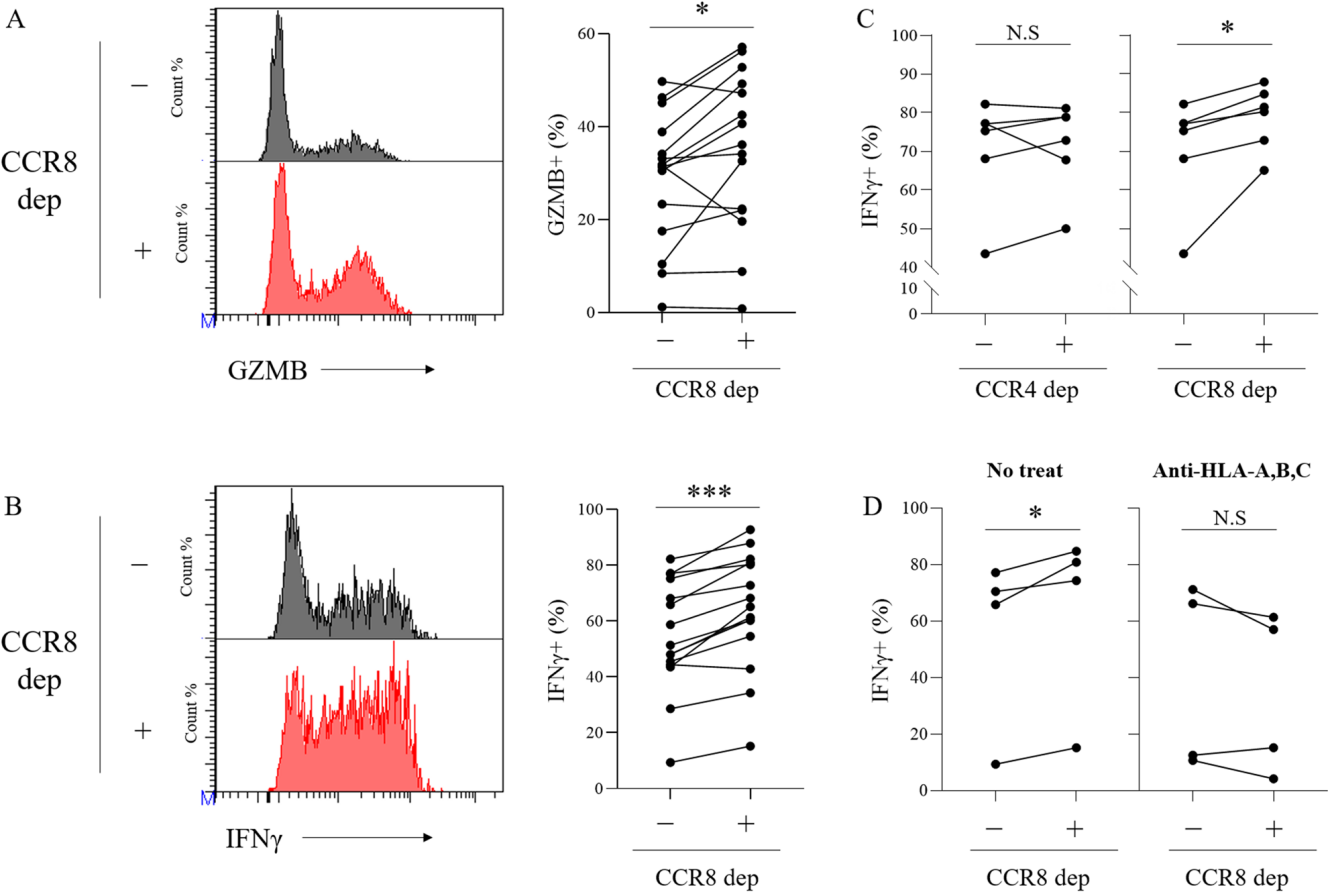
Impact of CCR8+ cell depletion on CD8 T cells in lung tumors. CCR8+ cells were magnetically removed from TICs of lung cancer and cultured with IL-2 and CpG. After 5 days, CD8 T cells in TICs were detected by flow cytometry. (**A**) Representative flow cytometric data (left) and the percentage of GZMB-expressing cells (right) in CD8T cells (gated on live CD45+ CD3+ CD8+ cells) are shown (n = 15). (**B**) Representative flow cytometric data (left) and percentage of IFNγ-producing cells (right) in CD8 T cells after PMA/ionomycin stimulation are shown (n = 15). (**C**) Percentages of IFNγ-producing cells in cultured CD8 T cells after CCR8+ cell and CCR4+ cell depletion (n = 5). (**D**) After depletion of CCR8+ cells, TICs were cultured in the presence of anti-HLA-A,B,C antibody for 5 days. Percentages of IFNγ-producing cells in CD8 T cells were detected by flow cytometry (n = 4). Lines connect data from the same donor samples. Statistical significance was determined by paired t-test (*p ≤ 0.05; **p < 0.01; ***p < 0.001).

We next examined whether depletion of CCR8+ Tregs could eliminate immunosuppression, leading to activation of CD8 T cells. We confirmed that CCR8+ cell depletion using MicroBeads reduced FOXP3+ Tregs but not FOXP3− conv CD4 T cells (Supplementary Fig. S4). To evaluate the influence of CCR8+ cell depletion on CD8 T cell functions, TICs with or without CCR8+ cell depletion were cultured with IL-2 and CpG for 5 days, and the expression of functional molecules in CD8 T cells was measured by flow cytometry (n = 15) (Fig. 5A, B). Depletion of CCR8+ cells led to an increased frequency of GZMB-expressing CD8 T cells and IFNγ-producing CD8 T cells upon PMA/ionomycin stimulation. Frequency of tumor necrosis factor-α (TNFα) -producing CD8 T cells was increased, too (data not shown). In contrast, the depletion of CCR4+ cells had no effect on IFNγ-producing CD8 T cells (n = 5) (Fig. 5C). To investigate the involvement of MHC class I in the suppression mechanism by CCR8+ Tregs, we added anti-HLA-A,B,C antibody in TIC culture after CCR8+ cell depletion. The results showed that the effect of CCR8+ cell depletion was canceled by treatment with anti-HLA-A,B,C antibody (n = 4) (Fig. 5D).

### Induced CCR8+ Tregs highly suppress CD8 T cell cytotoxicity

**Figure 6:**
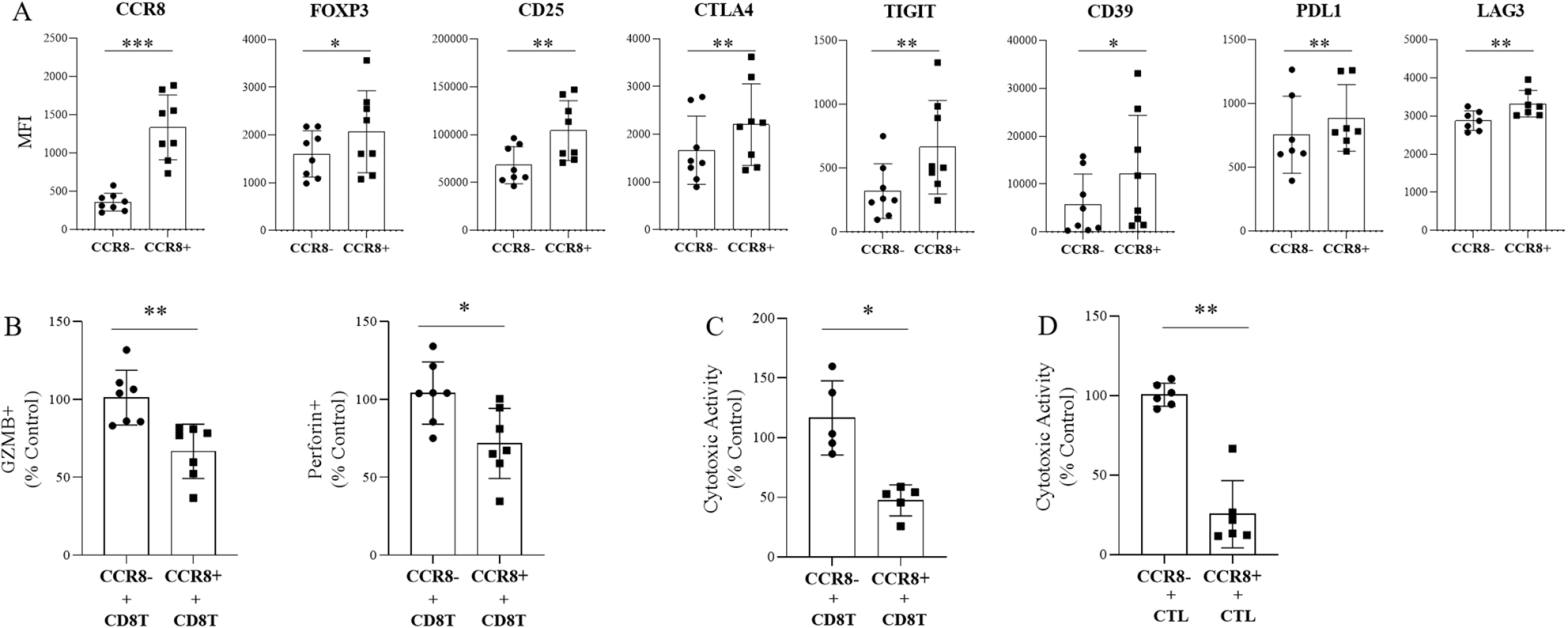
Suppressive effect of induced CCR8+ Tregs on CTL function. Tregs were purified from healthy donors and expanded. (**A**) Expression of Treg-related molecules on induced CCR8+ Tregs and CCR8− Tregs was analyzed by flow cytometry (n = 8). (**B**) CCR8+ Tregs and CCR8− Tregs were sorted and co-cultured with CD8 T cells prepared from healthy donor PBMCs in the presence of anti-CD3 antibody and APCs for 4 to 6 days. The expressions of GZMB and perforin in CD8 T cells were detected by flow cytometry (n = 7). The values were normalized to the percentage of GZMB or perforin expression in CD8 T cells cultured without Tregs set as 100%. (**C**) Antigen non-specific cytotoxicity of CD8 T cells after co-culture with Tregs was evaluated. CD8 T cells were co-cultured with anti-CD3scFv-expressing BALL-1 cells (aCD3-BALL-1 cells) (E:T ratio = 1:1) for 24 h; dead target cells were detected as propidium iodide (PI) + cells by flow cytometry (n = 6). The cytotoxic activities were calculated and normalized to the value of PI+ aCD3-BALL1 cells cultured with CD8 T cells without Tregs set as 100%. (**D**) Antigen-specific cytotoxicity of CTLs after co-culture with Tregs was evaluated. Mart-1 tetramer–positive CTLs were co-cultured with sorted CCR8+ Tregs or CCR8− Tregs for 4 to 6 days and then co-cultured with SK-MEL-5 cells (E:T ratio = 1:1) for 24 h. After lymphocytes were removed, WST assay was performed (n = 7). The cytotoxic activities were calculated and normalized to the cell viability of SK-MEL-5 cells cultured with CTLs without Tregs set as 100%. Statistical significance was determined by paired t-test (*p ≤ 0.05; **p < 0.01). Data are shown as mean values ± SD.

Next, we directly evaluated the suppressive potential of CCR8+ Tregs on CD8 T cell function. To obtain sufficient amounts of Tregs for functional assay, CCR8+ Tregs were induced and expanded from healthy donor PBMCs by anti-CD3/CD28 stimulation using Treg Expander Beads. We confirmed that expanded Tregs expressed FOXP3 and had suppressive function against effector T cell proliferation (Supplementary Fig. S5). Approximately 40% of the expanded Tregs expressed CCR8 (data not shown) and Treg-related molecules were expressed at higher levels in the CCR8+ fraction than the CCR8− fraction (n = 8) (Fig. 6A) as with tumor-infiltrating Tregs.

We sorted CCR8+ Tregs and CCR8− Tregs and examined the effects on CD8 T cell cytotoxicity using two models. To examine antigen non-specific cytotoxicity, sorted CCR8+ Tregs or CCR8− Tregs were co-cultured with CD8 T cells from healthy donor PBMCs and stimulated by anti-CD3 antibody in the presence of APCs. CD8 T cells co-cultured without Tregs served as controls. After 4 to 6 days of culture, we observed downregulation of GZMB and perforin expression in CD8 T cells co-cultured with CCR8+ Tregs (n = 7) (Fig. 6B). Antigen non-specific cytotoxicity of CD8 T cells was evaluated using membrane-bound anti-CD3scFv-expressing BALL-1 (aCD3-BALL1) cells (n = 6) (Fig. 6C). The results showed that the cytotoxicity against aCD3-BALL1 cells was suppressed only in CD8 T cells co-cultured with CCR8+ Tregs but not those cultured with CCR8− Tregs.

To examine antigen-specific cytotoxicity, sorted CCR8+ Tregs or CCR8− Tregs were co-cultured with Mart-1 tetramer-positive CTLs. CTLs co-cultured without Tregs served as control. After 4 to 6 days of culture, antigen-specific cytotoxicity of CTLs was evaluated using SK-MEL-5 cells (n = 7) (Fig. 6D). CCR8+ Tregs dramatically suppressed cytotoxic activity of CTLs, but CCR8− Tregs did not.

### Targeting CCR8 has anti-tumor effects in a murine model of lung cancer

**Figure 7:**
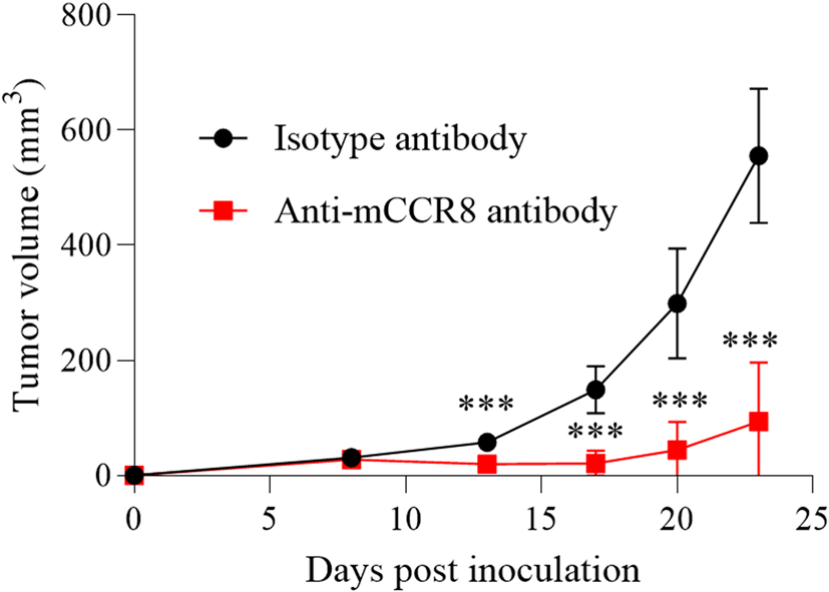
Anti-tumor effect of anti-CCR8 antibody in murine model of lung cancer. H-2 Kb-LLC cells were intradermally inoculated into C57BL/6 mice. At 3 and 8 days after tumor inoculation, the mice received intravenous injections of anti-CCR8 antibody or isotype control antibody (100 µg per mouse). Average tumor volumes are shown (n = 10 for each group). Statistical significance was determined by Mann–Whitney U test (***p < 0.001). Data are shown as mean values ± SD.

We finally evaluated the potential anti-tumor activity of CCR8-targeted therapy in a murine model of lung cancer. MHC class I-expressing Lewis lung carcinoma (H-2Kb-LLC) cells were established and inoculated them into C57BL/6 mice. To completely eliminate CCR8+ cells, anti-CCR8 antibody was administered twice at early time points, day 3 and day 8 after tumor inoculation. In the model mice, we observed infiltration of CCR8+ Tregs in tumor tissue, and treatment of anti-CCR8 antibody resulted in Treg reduction (Supplementary Fig. S6). Tumor growth was inhibited by anti-CCR8 antibody administration (n = 10) (Fig. 7). Tumors completely regressed in 5 of 10 mice treated with anti-CCR8 antibody.

## Discussion

In this study, we performed a detailed investigation of CCR8+ Tregs in human lung cancer and demonstrated that they have highly suppressive functions against CD8 T cells. As has been reported in other cancer types^21–23^, we found that CCR8 was selectively expressed on tumor-infiltrating Tregs in human lung cancer, and CCR8 expression on other immune cells was very low, indicating that adverse events may not be a concern for CCR8-targeted therapy. Prognostic analysis revealed that high infiltration of CCR8+ Tregs, but not CCR8− Tregs (data not shown), was significantly associated with poor prognosis (*p* = 0.011).

Previous studies reported shorter overall survival in cancer patients with high CCR8 expression by RNA-seq analysis of breast cancer and lung cancer, and by immunohistochemical and flow cytometric analysis of bladder cancer^23^. Our study showed a similar result through flow cytometric analysis of CCR8-expressing Tregs in lung tumors. These findings indicate that cancer patients with a high infiltration of CCR8+ Tregs may be more likely to experience recurrence. We recently reported that mice that showed tumor regression with anti-CCR8 therapy rejected re-inoculated tumors because of the formation of memory T cells, whereas mice in which tumors were surgically removed failed to form memory T cells and thus showed tumor regrowth^19^. We speculated that patients with a high infiltration of CCR8+ Tregs in tumors may not form an immunological memory and are thus more liable to show tumor metastasis and a shorter DFS. However, the small sample size is a limitation of this study, and our findings should be confirmed in larger cohorts.

In the present study, expression profiling of lung tumor-infiltrating CCR8+ Tregs revealed three features. First, we observed increased expression of Treg-related molecules in CCR8+ Tregs compared with CCR8− Tregs. Second, the adaptive immunity, inflammatory response, and cytokine receptor pathways were upregulated in CCR8+ Tregs. Third, CCR8+ Tregs showed higher expression of various chemokines and chemokine receptors compared with CCR8− Tregs. High expression of Treg-related molecules in CCR8+ Tregs has been reported in several studies^18–20,23^. We found that CCR8+ Tregs in human lung cancer exhibited a similar suppressive expression profile to previous studies, and further revealed that they are in a strongly activated state and enhance chemokine signaling. CCR8+ Tregs may recruit effector T cells to their surroundings by producing various chemokines, leading to efficient suppression of this cell activation. Interestingly, CCR8+ Tregs highly express CCL22 and its receptor CCR4, as well as CCL20 and its receptor CCR6^25^, and thus CCR8+ Tregs might form a Treg accumulation site through the autocrine activity of the chemokine and its receptor, leading to the induction of an immunosuppressive environment.

In addition, we showed that CCR8+ Tregs have strong suppressive functions against other immune cells, especially CTLs, through three approaches. First, using public lung cancer mRNA expression databases, we revealed that CCR8-high tumors showed reduced T cell- and APC-related genes and significantly lower naïve and effector CD8 T cell scores compared with CCR8-low tumors. Although there was no significant difference in prognosis between the high and low CCR8 expression groups, probably because of the use of multicenter TCGA data, the results suggest that CCR8+ Tregs might suppress mobilization, differentiation, or activation of naïve/effector CD8 T cells. Effector T cell fractions were characterized by high expression of genes associated with cytotoxicity^24^, and thus these findings suggest that CTL activation might be suppressed, and that the anti-tumor activity might be decreased in cancers with high infiltration of CCR8+ Tregs. Second, we experimentally showed that depletion of CCR8+ cells from TIC culture enhanced CD8 T cell function such as GZMB and IFNγ expression, while depletion of CCR4+ cells did not induce CD8 T cell activation. CCR4 is known to be expressed on both Tregs and conv CD4 T cells^26^, and in our experiment, depletion of CCR4+ cells not only reduced Tregs but also conv CD4 T cells, which might play a positive role in anti-tumor immunity. Hence, CCR8+ cell depletion may have activated CD8 T cells more efficiently than CCR4+ cell depletion. In this assay, blocking MHC class I canceled the CD8 T cell activation by CCR8+ cell depletion. Tumor antigen is taken up and processed by APCs and presented to CTLs by MHC class I^11,27^. Blocking of MHC class I inhibits this process. Thus, CCR8+ Tregs were considered to suppress CD8 T cell activation via APCs. Third, we directly evaluated the immune suppressive function of CCR8+ Tregs in CTL cytotoxicity using CCR8+ Tregs induced from PBMCs. In the co-culture with CD8 T cells, only CCR8+ Tregs dramatically suppressed the cytotoxic ability of CD8 T cells in both the antigen non-specific model and the antigen-specific model. Interestingly, CCR8− Tregs did not suppress cytotoxicity in either model. We speculated that CCR8+ Tregs may be responsible for most Treg suppressive functions against CTL cytotoxicity. In these experiments, it took 4 days for CCR8+ Tregs to suppress CD8 T cell cytotoxicity, and CCR8+ Tregs did not suppress CD8 T cell cytotoxicity at earlier time points (data not shown). CCR8+ Tregs are likely to suppress the differentiation process of CD8 T cells to acquire CTL functions over the 4-day period. On the basis of the results from these three approaches, we concluded that CCR8+ Tregs strongly suppressed CD8 T cell effector functions for cancer immunity.

There have been several reports showing that human CCR8+ Tregs are highly suppressive on the basis of their expression profiles, but only a few reports directly demonstrated their suppressive function against CD8 T cell activity. One report by Wang et al.^23^ revealed that CD8 T cells in muscle-invasive bladder cancer with high levels of CCR8+ Tregs displayed decreased expression of effector molecules (IFNγ and TNFα) and elevated expression of inhibitory receptors (PD-1 and TIGIT). Although we did not analyze such a detailed CD8 T cell profile by flow cytometry in the lung tumors of the high and low CCR8+ Treg groups, our GSVA analysis using TCGA dataset of lung cancer had similar results to the study by Wang et al*.* in terms of effector CD8 T cells^23^. Most likely, the results in exhausted CD8 T cells differed depending on whether the analysis was performed on whole tumors or only in CD8 T cells. Wang et al*.* also showed that blockade of CCR8 with CCR8 neutralizing antibody in ex vivo tumor culture for 12 h promoted Treg destabilization and led to significant upregulation of IFNγ and TNFα in CD8 T cells^23^. Our study demonstrated that CCR8+ Treg depletion using MicroBeads markedly enhanced not only IFNγ and TNFα expression but also that of the cytotoxicity molecule GZMB via APCs during 5-day ex vivo culture of lung TICs. These results estimated the effect of anti-CCR8-depleting antibody in humans. Furthermore, we successfully induced, isolated, and co-cultured CCR8+ Tregs with CD8 T cells and demonstrated that only CCR8+ Tregs exhibited the ability to suppress the cytotoxic activity of CTLs, whereas CCR8− Tregs could not suppress it. This is first report to reveal the direct suppressive functions of CCR8+ Tregs against CD8 T cells using human lung cancers and induced human Tregs. However, the effects on APCs, conv CD4 T cells, and other effector cells were not evaluated, and this is an issue for future study.

Several mechanisms of Treg suppressive functions in CTLs have been identified, such as the production of suppressive cytokines, IL-2 consumption, the APC-mediated pathway via CTLA-4, TIGIT, and LAG3, the metabolite-related mechanism via CD39/CD73, contact-dependent suppression by PD-L1, and other mechanisms^9,10,12,28–30^. In this study, the results of CCR8+ cell depletion among TICs showed the requirement of APCs for the suppression of CD8 T cells by CCR8+ Tregs. We observed higher expression of CTLA-4, TIGIT, and LAG3 in CCR8+ Tregs than in CCR8− Tregs, and therefore we speculated that the CCR8+ Treg suppressive functions exerted via APCs by these molecules are important for suppressing CD8 T cell function. In addition, a Treg suppressive pathway independent of APCs was also observed. Induced CCR8+ Tregs suppressed the antigen-specific cytotoxicity of CTLs, even in the absence of IL-2 and APCs. We surmised that the production of inhibitory cytokines such as TGFβ and IL-10 or the expression of CD39 and PD-L1 are important for suppressing CTL cytotoxicity under these conditions. Further analysis is necessary to investigate the most important mechanism of CCR8+ Tregs for suppressing CTL functions and why CCR8− Tregs do not show these effects.

Finally, we revealed the therapeutic potential of targeting CCR8 for cancer treatment using a murine model of lung cancer. Treatment with anti-CCR8 antibody reduced Tregs in tumor tissue and showed a remarkable anti-tumor effect including complete response. These results suggest that CCR8-targeted therapy is effective for lung cancer in vivo. From the results of ex vivo culture of human lung TICs (Fig. 5), CD8 T cell activation was observed with the removal of small amounts of Tregs. This indicates that even if the depletion efficiency is slightly low, it can still be effective. In this study, we used a subcutaneous transplantation model because of the difficulty in conducting stable orthotopic tumor transplantation, but the evaluation of CCR8-targeted therapy in an orthotopic lung cancer model is another issue for future study.

In conclusion, we revealed the pathophysiological characteristics of CCR8+ Tregs in human lung cancers and their inhibitory effect on CD8 T cells. We found that CCR8+ Tregs are responsible for the suppressive function against CTL cytotoxicity. We also revealed the anti-cancer efficacy of CCR8-targeted therapy using a murine lung cancer model. We provide evidence that CCR8-targeted therapy may be effective for the treatment of lung cancer. Several CCR8-targeted drugs are currently in development, and we are also planning a clinical study of anti-human CCR8 antibody for human cancer. Together, these findings may help to provide evidence and a foundation for the development of CCR8-targeted immunotherapy for cancers including lung carcinoma.

## Materials and methods

### Human samples

We obtained 50 fresh lung tumor tissues and 11 peripheral blood samples from lung cancer patients during primary surgical treatment from May 2017 to November 2020 at Osaka University Hospital. The clinical characteristics of the patients are summarized in Supplementary Table S1. Patients did not receive any neoadjuvant therapy. Peripheral blood samples from 7 healthy donors were also obtained. All participants provided written informed consent before sampling. This study was conducted in accordance with the Declaration of Helsinki and was approved by the Institutional Ethics Committee of Osaka University Hospital (#13266-15, #8226-10).

### Blood and tissue sample preparation

PBMCs were purified by gradient density centrifugation using Lymphoprep (Axis Shield, Dundee, UK). To prepare tissue-infiltrating cells in tumor tissues, tumor-adjacent normal tissues and lymph nodes, patients tissue samples were minced using surgical scalpels and further enzymatically dissociated using the human Tumor Dissociation Kit (Miltenyi Biotec, Bergisch Gladbach, Germany) and gentleMACS Dissociator (Miltenyi Biotec) according to the manufacturer’s protocol. Cell suspensions were filtered through a 70-mm cell strainer and isolated by Percoll (GE Healthcare, Tokyo, Japan) gradient centrifugation.

### Flow cytometry

Cells were stained by fluorescence-conjugated antibodies after dead cell staining (fixable viability dye; Thermo Fisher Scientific, Waltham, MA, USA) and Fc receptor blocking (Human TruStain FcX; BioLegend, San Diego, CA, USA). The Foxp3/Transcription Factor Staining Buffer Kit (Thermo Fisher Scientific) was used for intracellular staining. The antibody list for flow cytometry is shown in Supplementary Table S3. Stained cells were detected by LSR Fortessa (BD Bioscience, San Jose, CA, USA).

### RNA-sequencing and data processing

One million CCR8+ or CCR8− Tregs (CD45+ CD3+ CD4+ CD45RA-CD25^high^) cells were sorted from three lung tumors by FACS Aria II (BD Bioscience). RNA was extracted and purified using TRIzol Reagent (Thermo Fisher Scientific) and miRNeasy Micro Kit (Qiagen, Germany). Library preparation was performed using RNA with the Ion Total RNA-Seq Kit v2 (Thermo Fisher Scientific) and sequencing was performed by Ion Proton in triplicate. Sequencing data were mapped to mm9 with TopHat2 (version 2.0.11). The count data are shown in Supplementary Table S4. For analysis of DEGs between CCR8+ Tregs and CCR8− Tregs, tag counts obtained by HT-seq (version 0.6.1) and the data were normalized using DESeq2 package in R software (version 4.1.0). We performed GO enrichment analysis for biological process terms in the SOM bidimensional space using DAVID (https://david.ncifcrf.gov/home.jsp). The top 100 DEGs with the lowest p-value among CCR8+ Treg upregulated genes were used for terms ranking and selection of DAVID.

### The Cancer Genome Atlas (TCGA) analysis

We downloaded TCGA Pan-Cancer Atlas mRNA expression data including lung adenocarcinoma and lung squamous cell carcinoma data from cBioPortal (https://www.cbioportal.org). The mRNA expression data were normalized by Expectation–Maximization method. GSVA was performed according to the method by Hänzelmann et al.^30^ using R package (version 4.1.0). According to Guo et al*.*, signature genes of the CD8-C1-LEF1 fraction, CD8-C3-CXCR1 fraction and CD8-C6-LAYN fraction were used as naïve, effector and exhausted CD8 T cell gene sets, respectively^23^. We added the *CD8A* gene to each signature gene set. The gene sets are shown in Supplementary Table S5.

### Ex vivo culture of tumor-infiltrating cells after depletion of CCR8+ cells

CCR8+ cells were removed from freshly isolated TICs by PE-conjugated anti-human CCR8 antibody (L263G8; BioLegend) and anti-PE MicroBeads (Miltenyi Biotec). For the depletion of CCR4+ cells, PE-conjugated anti-human CCR4 antibody (L291H4; BioLegend) was used. PE-conjugated isotype control antibody was used as control. After depletion, the cells were cultured with 10 U/ml human IL-2 (Immunace, Shionogi, Osaka, Japan) and ODN 2006 (Thermo Fisher Scientific) in RPMI-1640 (Nacalai Tesque, Japan) with 10% fetal bovine serum (FBS) (HyClone; Thermo Fisher Scientific) at 37 °C under 5% CO_2_ for 5 days. To block MHC class I, anti-human HLA-A,B,C antibody (W6/32; BioLegend) was added in the culture. Granzyme B (GZMB) expression in CD8+ T cells was detected by flow cytometry. IFNγ-producing cells in CD8+ T cells were also analyzed by flow cytometry after 4 h stimulation with phorbol 12-myristate 13-acetate (PMA; 50 ng/ml) and ionomycin (1 µM) (Sigma-Aldrich, St. Louis, MO, USA) in the presence of BD GolgiStop (BD Bioscience).

### Induction of CCR8+ Tregs from PBMCs

CD4 T cells were purified from healthy donor PBMCs by the human CD4 T Cell Isolation Kit (Miltenyi Biotec). Then, Naïve Tregs (CD4+ CD127^low^CD25+ CD45RA+) were sorted by FACSAria II (BD Bioscience) and expanded by Dynabeads Human Tregs Expander (Thermo Fisher Scientific) in accordance with the manufacturer’s instructions. After expansion, the CCR8+ Tregs or CCR8− Tregs were sorted by MACS Quant Tyto (Miltenyi Biotec).

### Mart-1 CTL induction

PBMCs from a HLA-02:01-positive donor obtained from Cellular Technology Limited (CLE, OH, USA) were cultured in AIM-V medium (Thermo Fisher Scientific) with 10% FBS containing 02;01-Mart-1 peptide (1 µg/ml) (Medical & Biological Laboratories, Tokyo, Japan) and IL-2 (50 U/ml; Immunace). After 14 days of culture, cells were stained with CD8a and HLA-A*02:01 Mart-1 Tetramer-ELAGIGILTV (Medical & Biological Laboratories), and Mart-1 tetramer–positive cells were sorted using a MACS Quant Tyto. Sorted cells were cultured with human IL-15 (100 U/ml; Miltenyi Biotec) for an additional 14 days. The Mart-1 tetramer–positive cells were sorted again and stored in an N_2_ bank. We confirmed that prepared cells had antigen-specific cytotoxicity against Mart-1 expressing target cells but not target cells that do not express Mart-1.

### Cytotoxicity assay of CD8 T cells after co-culture with Tregs

For antigen-nonspecific cytotoxicity assay, CD8+ cells were isolated from healthy donor PBMCs by CD8 MicroBeads (Miltenyi Biotec). PBMCs depleted of CD8+ and CD4+ cells by MicroBeads (Miltenyi Biotec) were used as APCs. CD8+ cells (10^4^ cells), APCs (10^5^ cells) and CCR8+ Tregs or CCR8− Tregs (10^4^ cells) were co-cultured with anti-human CD3 antibody (1 µg/ml) (OKT3; BioLegend) in RPMI-1640 with 10% FBS. After 4–6 days of culture, a cytotoxicity assay targeting membrane-bound anti-CD3scFv-expressing BALL-1 (aCD3-BALL1) cells was performed. aCD3-BALL1 cells were previously established^31^. The percentage of CD8+ cells in the co-culture was calculated using MACS Quant (Miltenyi Biotec), and cell suspensions of 2 × 10^3^ CD8+ T cells were co-cultured with 2 × 10^3^ aCD3-BALL1 cells, which were labeled with CytoTell Blue (AAT Bioquest, San Jose, CA, USA). On the next day, the cultured cells were stained with propidium iodide (PI) (BioLegend) and the proportion of PI+ cells among CytoTell Blue-labeled aCD3-BALL1 cells was analyzed by MACS Quant. The cytotoxicity was calculated and normalized to the cell viability of PI+ aCD3-BALL1 cells cultured with CD8 T cells without Tregs set as 100%.

For antigen-specific cytotoxicity assay, thawed Mart-1 CTLs (10^4^ cells) were cultured for 4–6 days with CCR8+ Tregs or CCR8− Tregs (10^4^ cells) in AIM-V medium with 10% FBS. After 4–6 days of culture, cytotoxicity assay targeting SK-MEL-5 cells (American Type Culture Collection, Manassas, MA, USA) was performed. The percentage of CD8+ cells in the co-culture were calculated using MACS Quant, and cell suspensions of 2 × 10^3^ CD8 T cells were co-cultured with 2 × 10^5^ SK-MEL-5 cells pre-cultured from the previous day. On the following day, cell viability was evaluated using the Cell Counting Kit-8 (Dojindo, Japan). The cytotoxicity was calculated and normalized to the cell viability of SK-MEL-5 cells cultured with CD8 T cells without Tregs set as 100%.

### Establishment of H-2Kb-expressing LLC cells

The H-2Kb cDNA was synthesized from mRNA of C57BL/6 mouse spleen and inserted into the pCMV6-AC-Myc-DDK-IRES-GFP-Puro Mammalian Expression Vector (ORIGENE, USA, Rockville, MD, USA). LLC cells, purchased from American Type Culture Collection, were transfected with pCMV6-AC-Myc-DDK-IRES-GFP-Puro-H-2Kb by Lipofectamine 3000 Reagent (Thermo Fisher Scientific). H-2Kb-LLC cells were purified by FACS Aria II (BD Bioscience) three times.

### In vivo anti-tumor study in the mouse H-2Kb-LLC model

Six-week-old female wild-type C57BL/6 mice were obtained from CLEA-Japan (Tokyo, Japan). Mice were kept under specific pathogen free conditions and provided with food and water ad libitum. The experiments were conducted in compliance with the Act on Welfare and Management of Animals in Japan and the Guide for the Care and Use of Laboratory Animals and in accordance with the protocol approved by the Institutional Animal Care and Use Committee of Shionogi & Co., Ltd., which is accredited by AAALAC International. The study was carried out in compliance with Animal Research Reporting of In Vivo Experiments (ARRIVE) guidelines.

H-2Kb-LLC cells (2 × 10^5^ cells) were intradermally inoculated into C57BL/6 mice. At 3 and 8 days after tumor inoculation, the mice were intravenously injected with anti-mouse CCR8 antibody (100 µg/mouse) (SA214G2; BioLegend) or mouse IgG2b, κ isotype control antibody (27–35; BioLegend) (n = 10 for each group). Tumor volume was monitored and calculated (mm^3^) as follows: major axis (mm) × minor axis (mm) × minor axis (mm)/2. All tumor-bearing mice were euthanized according to institutional animal care guidelines on the basis of tumor size, body weight or general condition. Euthanasia was performed by isoflurane inhalation followed by cervical dislocation.

### Statistical analysis

GraphPad Prism Version 8 and Excel software were used for statistical analysis. ANOVA was used for group comparisons, using a Dunn post hoc multiple comparison test. The difference between the two groups was assessed using Student’s t-test or two-tailed paired t-test. The disease-free survival DFS rate were estimated using the Kaplan–Meier method and compared by the log-rank test. The Mann–Whitney U test was used for results from the in vivo study. A value of *p* ≤ 0.05 were considered significant.

## Supplementary Information

Supplementary Figures.

Supplementary Tables.

Supplementary Table 4.

Supplementary Table 5.

## Data Availability

The human RNA-sequence data have been deposited in the DNA Data Bank of Japan under accession number JGAS000454. Data generated during this study are available from the corresponding author on reasonable request.
