# Supplementary material for: The impact of CCR8+ regulatory T cells on cytotoxic T cell function in human lung cancer
Source: Sci Rep. 2022 Mar 30;12:5377. doi: 10.1038/s41598-022-09458-5 (PMC8967908; doi:10.1038/s41598-022-09458-5)
Supplement: Supplementary file 1 — Supplementary Figures. [file 41598_2022_9458_MOESM1_ESM.pptx]

## Slide 1
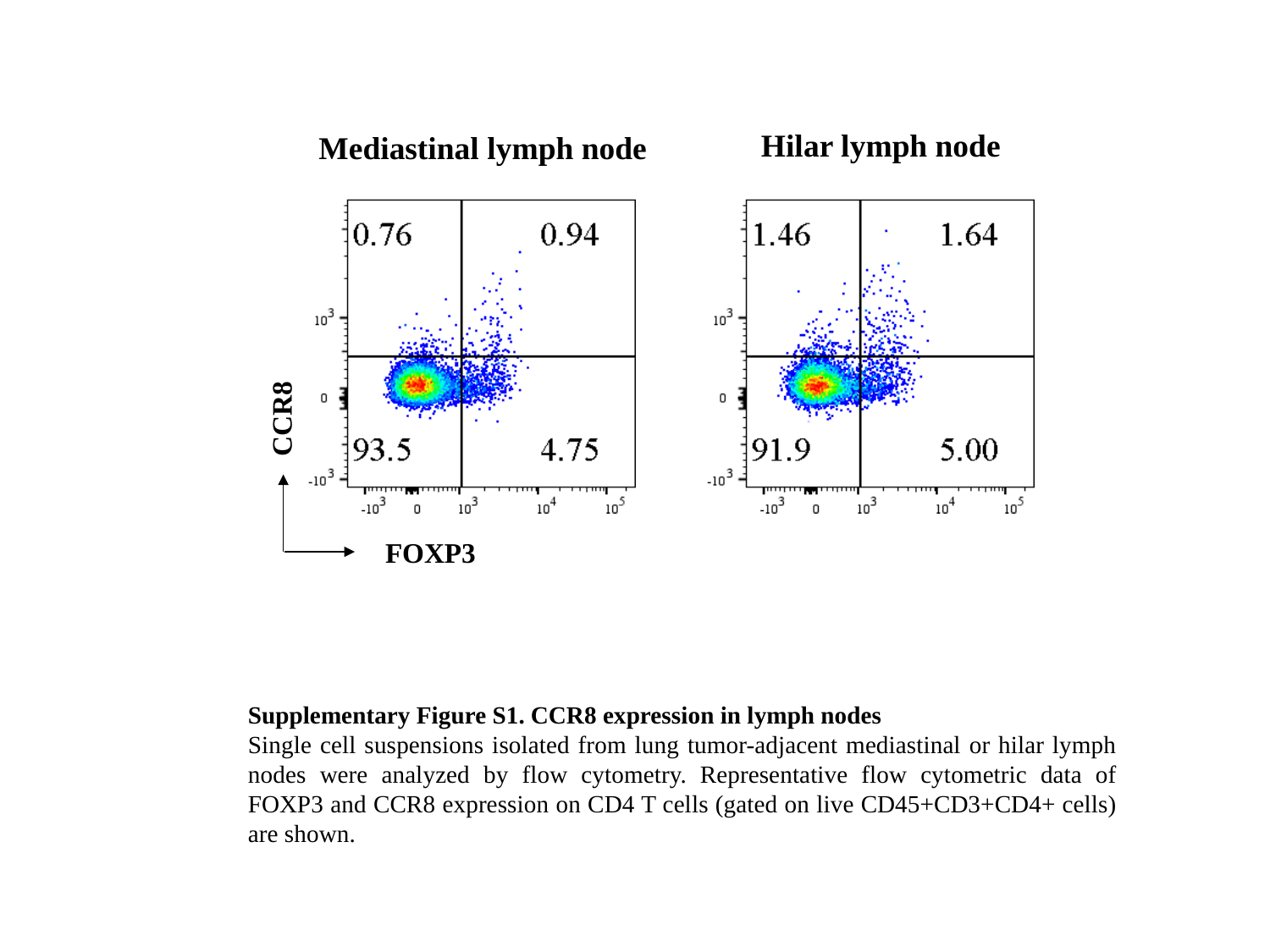

Hilar lymph node
Mediastinal lymph node
CCR8
FOXP3
Supplementary Figure S1. CCR8 expression in lymph nodes
Single cell suspensions isolated from lung tumor-adjacent mediastinal or hilar lymph nodes were analyzed by flow cytometry. Representative flow cytometric data of FOXP3 and CCR8 expression on CD4 T cells (gated on live CD45+CD3+CD4+ cells) are shown.

## Slide 2
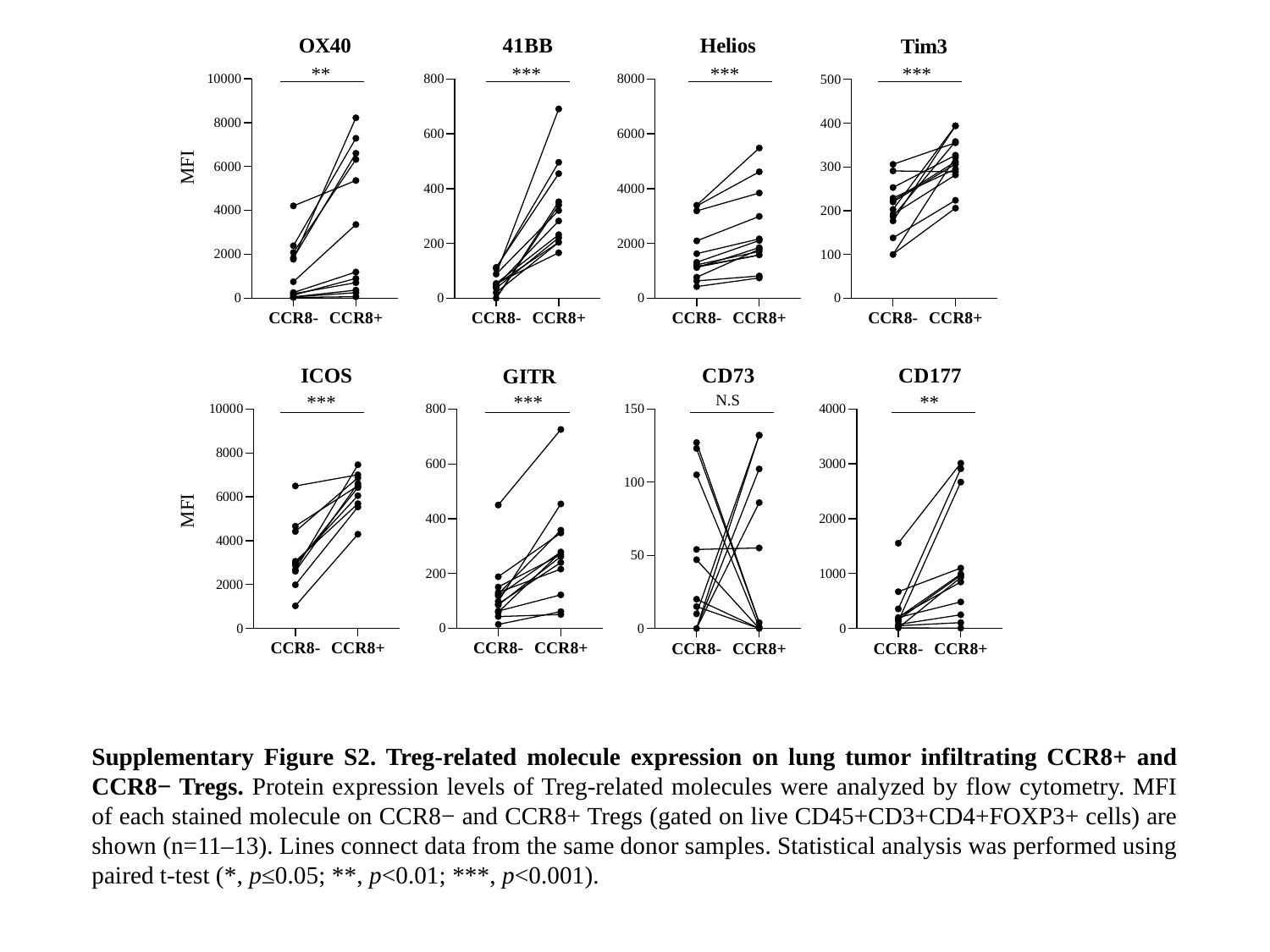

**
***
***
***
MFI
***
***
N.S
**
MFI
Supplementary Figure S2. Treg-related molecule expression on lung tumor infiltrating CCR8+ and CCR8− Tregs. Protein expression levels of Treg-related molecules were analyzed by flow cytometry. MFI of each stained molecule on CCR8− and CCR8+ Tregs (gated on live CD45+CD3+CD4+FOXP3+ cells) are shown (n=11–13). Lines connect data from the same donor samples. Statistical analysis was performed using paired t-test (*, p≤0.05; **, p<0.01; ***, p<0.001).

## Slide 3
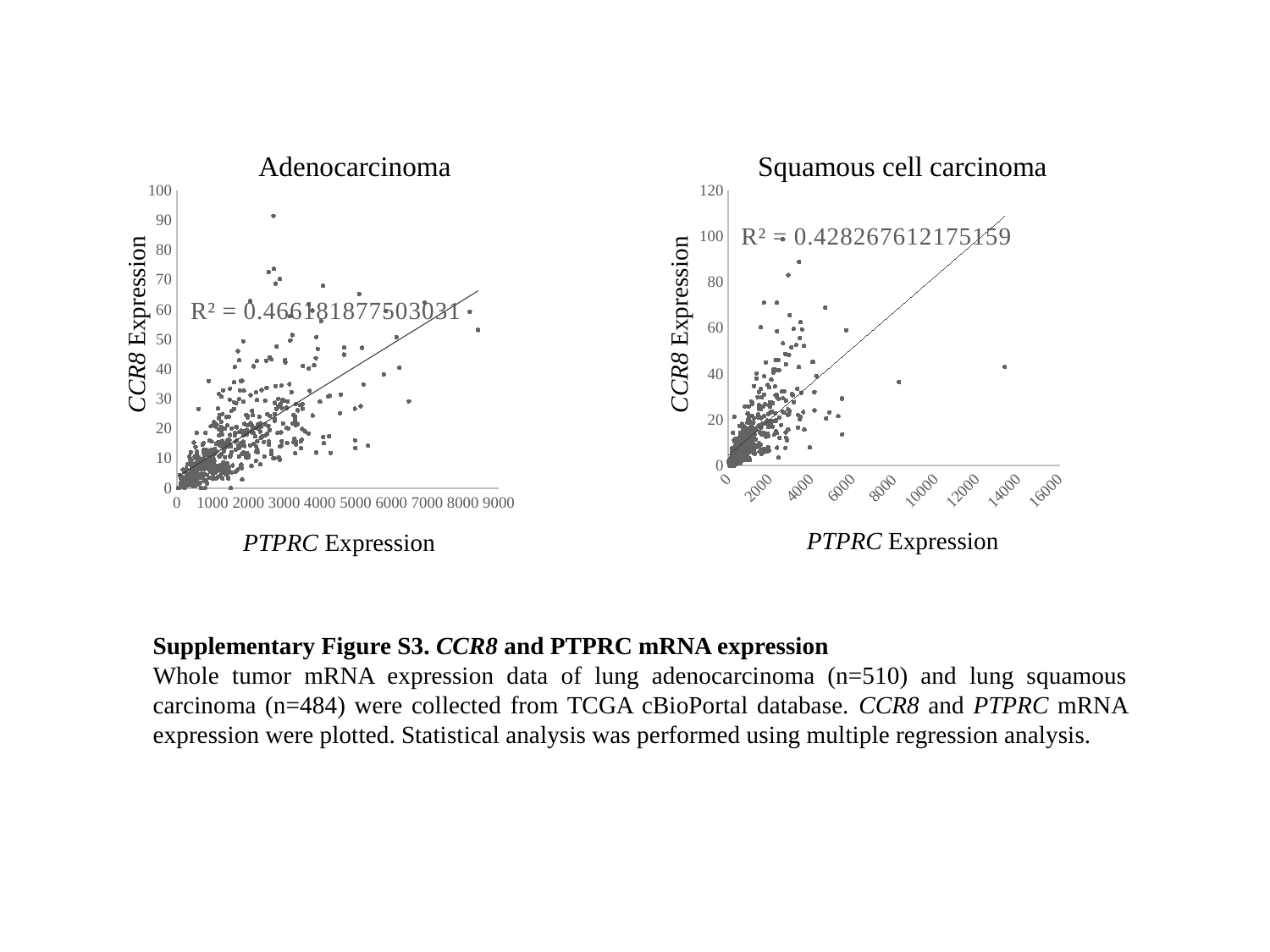

Adenocarcinoma
Squamous cell carcinoma
### Chart
| Category | |
|---|---|
### Chart
| Category | |
|---|---|CCR8 Expression
CCR8 Expression
PTPRC Expression
PTPRC Expression
Supplementary Figure S3. CCR8 and PTPRC mRNA expression
Whole tumor mRNA expression data of lung adenocarcinoma (n=510) and lung squamous carcinoma (n=484) were collected from TCGA cBioPortal database. CCR8 and PTPRC mRNA expression were plotted. Statistical analysis was performed using multiple regression analysis.

## Slide 4
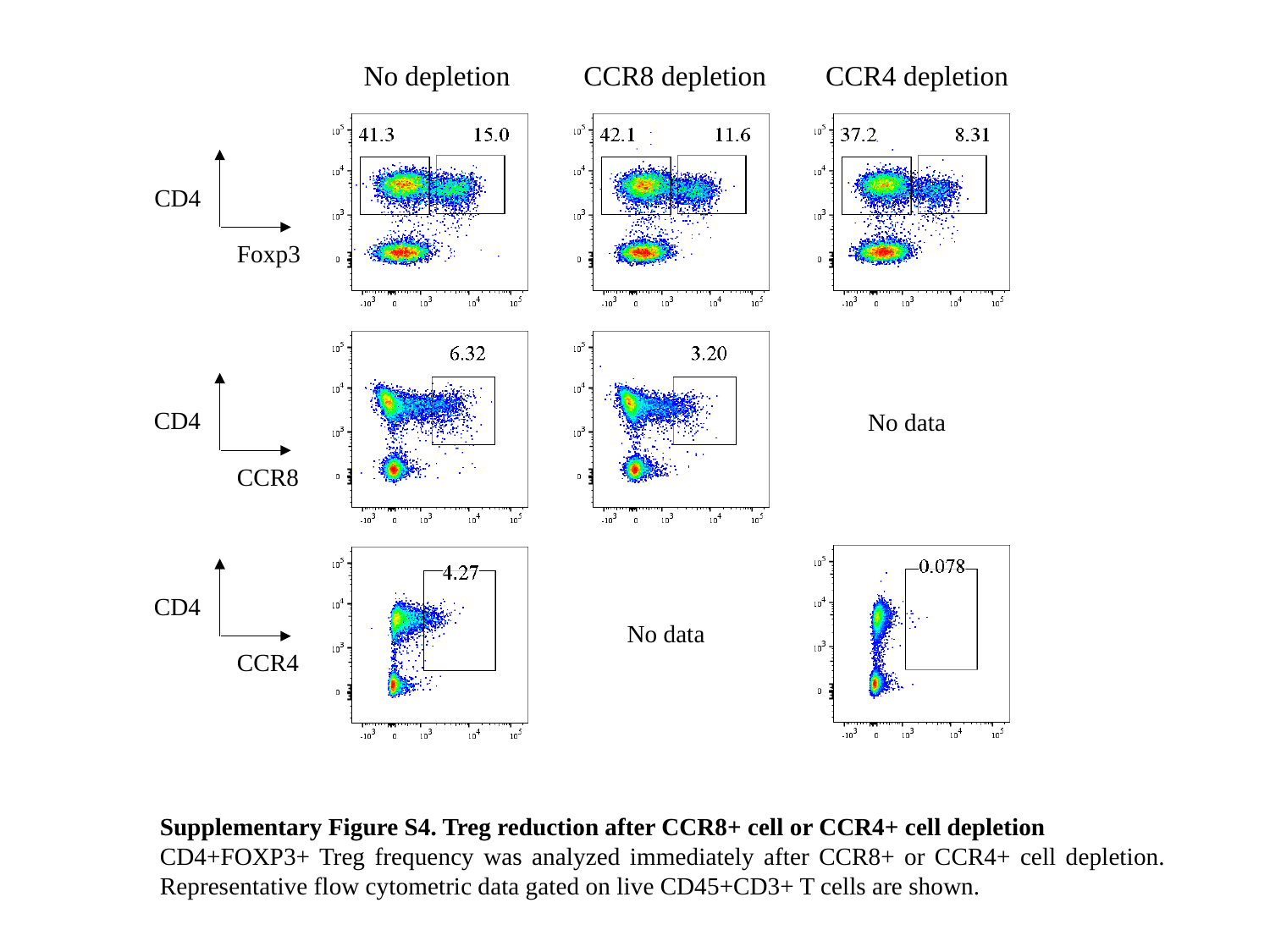

No depletion
CCR8 depletion
CCR4 depletion
CD4
Foxp3
CD4
No data
CCR8
CD4
No data
CCR4
Supplementary Figure S4. Treg reduction after CCR8+ cell or CCR4+ cell depletion
CD4+FOXP3+ Treg frequency was analyzed immediately after CCR8+ or CCR4+ cell depletion. Representative flow cytometric data gated on live CD45+CD3+ T cells are shown.

## Slide 5
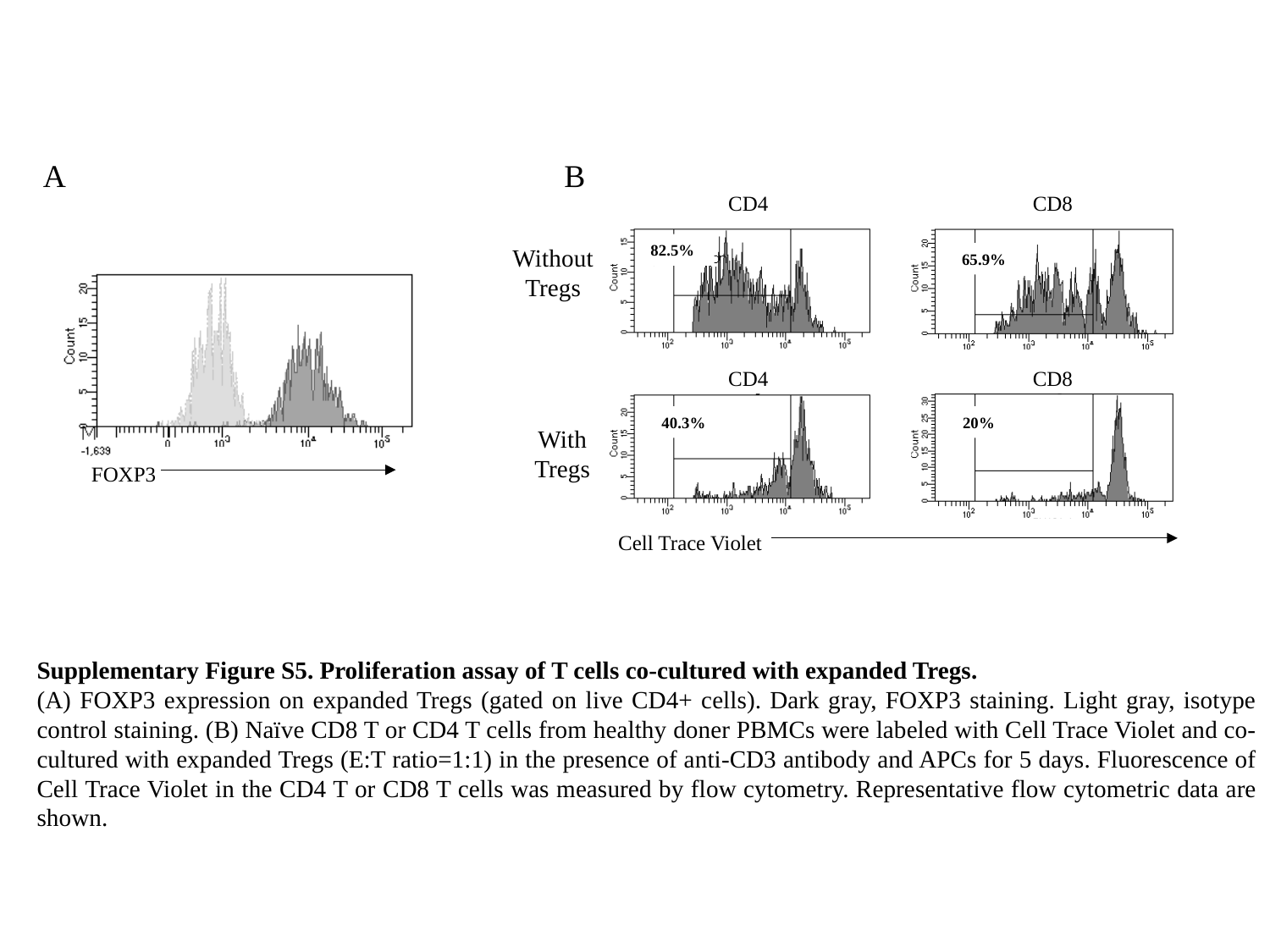

A
B
CD8
CD4
82.5%
Without
Tregs
65.9%
CD8
CD4
40.3%
20%
With
Tregs
Cell Trace Violet
FOXP3
Supplementary Figure S5. Proliferation assay of T cells co-cultured with expanded Tregs.
(A) FOXP3 expression on expanded Tregs (gated on live CD4+ cells). Dark gray, FOXP3 staining. Light gray, isotype control staining. (B) Naïve CD8 T or CD4 T cells from healthy doner PBMCs were labeled with Cell Trace Violet and co-cultured with expanded Tregs (E:T ratio=1:1) in the presence of anti-CD3 antibody and APCs for 5 days. Fluorescence of Cell Trace Violet in the CD4 T or CD8 T cells was measured by flow cytometry. Representative flow cytometric data are shown.

## Slide 6
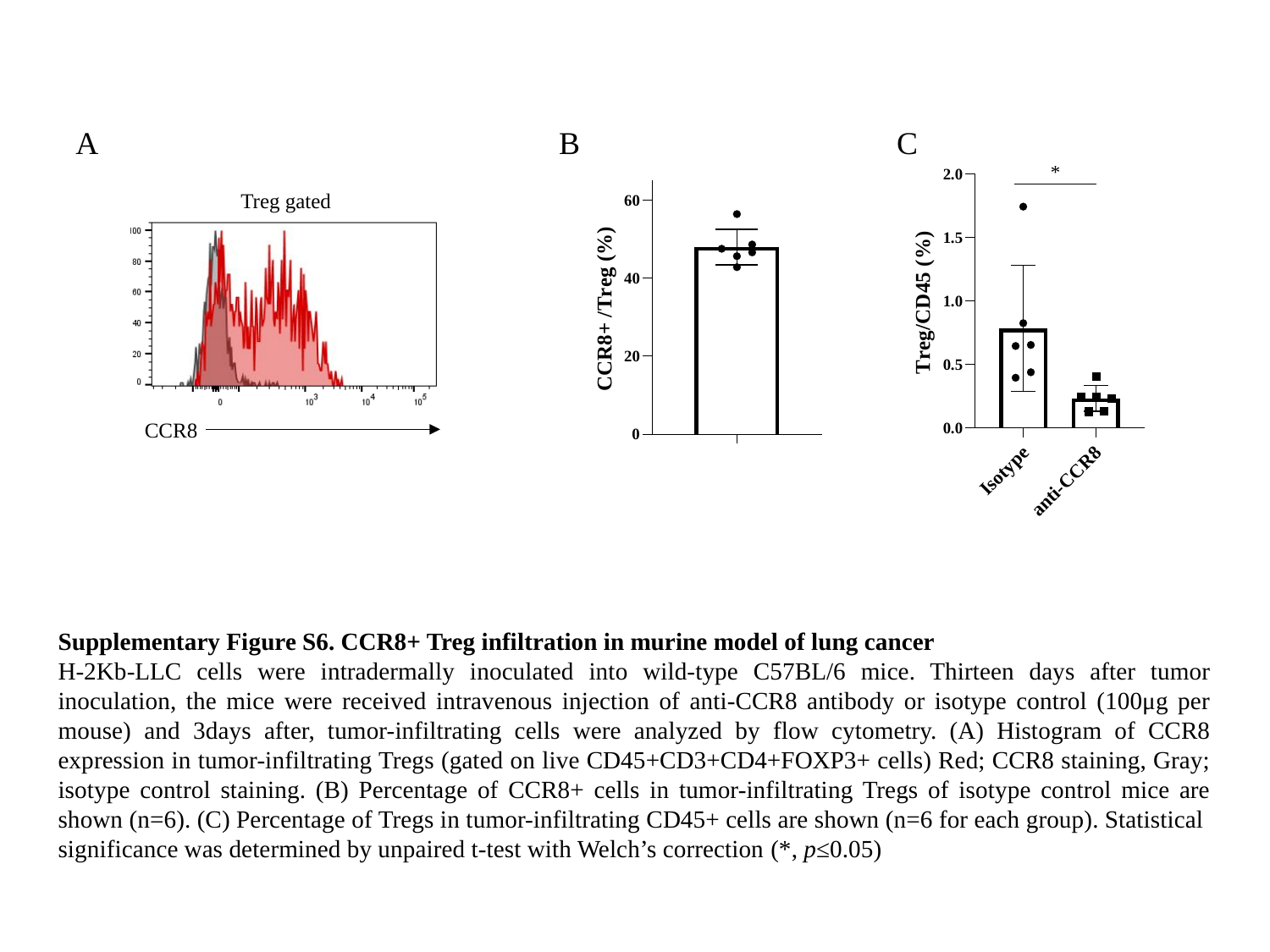

A
B
C
*
Treg gated
CCR8
Supplementary Figure S6. CCR8+ Treg infiltration in murine model of lung cancer
H-2Kb-LLC cells were intradermally inoculated into wild-type C57BL/6 mice. Thirteen days after tumor inoculation, the mice were received intravenous injection of anti-CCR8 antibody or isotype control (100μg per mouse) and 3days after, tumor-infiltrating cells were analyzed by flow cytometry. (A) Histogram of CCR8 expression in tumor-infiltrating Tregs (gated on live CD45+CD3+CD4+FOXP3+ cells) Red; CCR8 staining, Gray; isotype control staining. (B) Percentage of CCR8+ cells in tumor-infiltrating Tregs of isotype control mice are shown (n=6). (C) Percentage of Tregs in tumor-infiltrating CD45+ cells are shown (n=6 for each group). Statistical significance was determined by unpaired t-test with Welch’s correction (*, p≤0.05)
